# Circulating leukocyte gene expression responses to weaning and their association with growth in holstein calves

**DOI:** 10.1371/journal.pone.0349643

**Published:** 2026-05-27

**Authors:** Marta Sfulcini, Erminio Trevisi, Fiorenzo Piccioli-Cappelli, Andrea Minuti

**Affiliations:** 1 Department of Animal Science, Food and Nutrition (DIANA), Faculty of Agricultural, Food and Environmental Sciences, Università Cattolica del Sacro Cuore, Piacenza, Italy; 2 Romeo and Enrica Invernizzi Research Center for Sustainable Dairy Production of the Università Cattolica del Sacro Cuore (CREI), Piacenza, Italy; Michigan State University, UNITED STATES OF AMERICA

## Abstract

Weaning represents a critical transition in dairy calves, involving simultaneous dietary, social, and environmental changes. This study investigated how the weaning affects immune-related gene expression in circulating leukocytes of Holstein calves and whether post-weaning growth performance is associated with distinct immunometabolic responses. Forty female calves were retrospectively classified into two groups based on average daily gain (ADG) during the 10 days following complete weaning (from 60 to 70 days of age). Calves were fully weaned at 60 days of age using a step-down approach. Animals were categorized as high ADG (HIGH, n = 20) or low ADG (LOW, n = 20) Blood samples collected at 60 and 70 days were analyzed for plasma metabolites and leukocyte transcriptomic profiles targeting 37 genes involved in innate immunity, inflammation, oxidative stress, and metabolic regulation. Weaning induced a marked activation of innate immune pathways in all calves, as evidenced by increased expression of CD14, NFKB1, IL1β, TNFα, and genes related to cell adhesion and trafficking. However, LOW calves exhibited greater transcriptional responses, with higher expression of TLR2, CASP1, IL18, CD16, and markers of oxidative (SOD2, HSPA5) at 70 days, indicating a metabolic and transcriptional profile consistent with a greater physiological burden. Additionally, at 70 days, LOW calves had lower plasma glucose, insulin, triglycerides, and cholesterol, along with higher urea and creatinine, while β-hydroxybutyrate did not differ between groups, suggesting reduced energy availability and increased reliance on amino acid catabolism. These findings suggest that calves with lower post-weaning growth exhibit greater immune activation and altered metabolic status, highlighting variability in resilience to weaning stress. Understanding such differences may support the development of targeted nutritional or management strategies to improve early-life adaptation and long-term productivity in dairy systems

## Introduction

In dairy systems, weaning is defined as the end of milk feeding, after which the calf becomes fully dependent on solid feed and water [1]. This transition exposes calves to a combination of dietary, social, and environmental changes, including regrouping, housing adjustments, and altered feeding routines, which together make weaning a particularly challenging period [2,3]. Weaning distress has been associated with growth slumps, reduced resting, increased vocalization and activity, and a higher incidence of diseases, indicating that the welfare, health, and performance of calves can be markedly compromised during this transition [1].

Successful adaptation to weaning depends on metabolic and endocrine flexibility and on the development of a functional rumen able to ferment solid feed and absorb volatile fatty acids (VFA). Fermentable carbohydrates in starter feed promote butyrate and propionate production, stimulating rumen epithelial development and supporting post-weaning energy metabolism [4,5]. Inadequate adaptation to this dietary and digestive transition increases the risk of growth impairment, immune imbalance, and disease, with potential consequences for both short-term performance and long-term phenotype, underscoring the importance of early-life interventions to improve resilience to weaning stress [1].

During weaning, calves show increased vocalization, activity, and walking, indicating difficulty adapting to the new feeding regime [3,6]. These behavioral responses are accompanied by endocrine and inflammatory changes, including elevated cortisol, noradrenaline, and peripheral catecholamines, consistent with sympathetic activation [7]. Acute stress may trigger an acute-phase response (APR) through glucocorticoid release, leading to changes in cytokine and acute-phase protein production [8]. While short-term activation can be adaptive, prolonged or repeated stress may impair immune function and increase disease susceptibility [9,10].

The consequences of weaning management are not restricted to the immediate post-weaning period. Growing evidence shows that early-life nutritional and physiological experiences can influence long-term health, productivity, and metabolic programming [11–14]. Despite these known impacts on physiology and growth, to our knowledge no studies have specifically examined how the weaning process influences the expression of immune-related genes in circulating leukocytes, or whether differences in post-weaning growth performance are associated with distinct transcriptional profiles. A better understanding of these mechanisms may help identify early biomarkers of resilience to weaning stress and support the development of nutritional or management strategies aimed at improving post-weaning adaptation and long-term productivity. Therefore, the objective of this study was to investigate changes in immune- and stress-related gene expression in circulating leukocytes during the weaning transition and to evaluate their association with post-weaning growth performance. We hypothesized that weaning would induce activation of innate immune and stress-related pathways, and that calves with lower post-weaning growth would be associated with a more pronounced inflammatory and metabolic transcriptional response compared with calves with higher growth performance

## Materials and methods

### Animals Management and sampling

The study was prospectively evaluated and ethically approved by the Institutional Animal Welfare Body (OPBA) of the Università Cattolica del Sacro Cuore. The research was authorized by the Italian Ministry of Health (Authorization No. 130/2022-PR), in accordance with Italian legislation on animal experimentation (Legislative Decree No. 26 of March 4, 2014, implementing Directive 2010/63/EU). No anesthesia, euthanasia, or animal sacrifice was performed as part of the study.

The study was performed at Università Cattolica del Sacro Cuore dairy farm (Cerzoo, San Bonico, Piacenza, Italy). From September 2023 to January 2024, a total of 40 female Holstein calves were enrolled in the study. Within the first hour after birth, calves were separated from their dams and housed individually in straw-bedded hutches. After separation, they were cleaned, weighed, and had their navels disinfected with an oxytetracycline hydrochloride spray (Neo Spray Caf® Aerosol; Gellini S.p.a., Aprilia, Italy). During the first three hours of life, each calf was provided with 3 L of colostrum via nipple bottle. The colostrum had been previously collected, pooled to reach a Brix value of 28%, and frozen in 3 L portions. If a calf did not consume the entire amount on its own within one hour, the remaining colostrum was administered using an esophageal feeder (Speedy Drencher XL, Agri-Zoo San Marino srl, Domagnano, San Marino). Subsequent feedings at 12 and 24 hours of life consisted of transition milk. This milk was obtained from the second and third milkings of each calf’s own dam.

From 3 to 59 d, calves received milk replacer (MR) prepared at the rate of 130 g/L (23% crude protein, 18% fat, and 6.5% ash). The chemical composition of the MR is reported in Table 1.

**Table 1:** Nutrient composition (% of DM) of milk replacer (MR), calf starter, and calf concentrate.

Item	MR^1^	Calf starter^2^ until 60 d	Concentrate^3^ From 60 to 70 d
CP, % Crude fat, % Crude fiber, % Ash, % Na, % Ca, % P, %	23 18 0.0 6.1 0.34 0.82 0.59	21.5 2.3 6.7 6.5 0.02	17 2.9 10 5 0.2

^1^ MR, Emme erre 50®, Tredì Italia Srl, Italy

^2^ Calf starter, Baby progress®, Ferraroni Spa, Cremona, Italy

^3^ Concentrate, Born full fiber, Nutristar, Reggio Emilia, Italy

MR was given twice a day (0800 and 1630 h) using a nipple-bottle from day 2 until day 60 of age, when calves were fully weaned. Calves received 6 L from d 2–14 and 8 L from d 15–49 of age. The step-down weaning started gradually from the d 50, where calves received 6 L until 52 d, 4 L from 53 to 55 d, and 2 L from 56 to 60 d of age. From d 4–60, calf starter was offered *ad libitum*. The calf starter used was a commercial product prepared and pelleted (Calf starter, Baby progress®, Ferraroni Spa, Cremona, Italy). The chemical composition of the calf starter is reported in Table 1.

From d 60–70, calves were moved to a co-mingled pen bedded with straw and received *ad libitum* concentrate (Concentrate, Born full fiber, Nutristar, Reggio Emilia, Italy). The chemical compositions is reported in Table 1. All the animals were vaccinated at d 7 with Bovisil® Intranasal Rsp™ live (MSD Animal Health S.r.l) giving them 1 mL per nostril, and at days 21 and 42 with Bovilis Bovipast Rsp (MSD Animal Health S.r.l)..

The health status of calves was checked daily. None of the calves enrolled suffered from any acute health disorder during the entire period of the study. Body weight (BW) was measured at birth (d 0) and d 14, 28, 42, 60, 70, 90, 120 and 150. The measurements were recorded in the morning before feeding the MR and calf starter. The average daily gain (ADG) was calculated as the difference between BW values from two consecutive time points divided by the number of days between measurements. According to the ADG from 60 to 70 days of age, calves were retrospectively divided into two groups: low ADG (LOW; n = 20; 0.84 ± 0.05 Kg/d) and high ADG (HIGH; n = 20; 1.3 ± 0.05 Kg/d).

Blood samples were collected at 60 and 70 d of age into lithium-heparin tubes (BD Vacutainer®, Becton, Dickinson and Company, Franklin Lakes, NJ, USA) and PAXgene Blood RNA System tubes (Preanalytix, Hombrechtikon, Switzerland). Blood was withdrawn from the jugular vein before the morning MR meal. Lithium-heparin tubes were immediately cooled in an ice–water bath and then centrifuged at 3,500 × g for 15 min at 4°C. Plasma was harvested, divided into aliquots, and stored at −20°C. Plasma metabolites were analyzed at 37°C with an automated clinical analyzer (ILAB 650; Instrumental Laboratory) as previously described [15,16]. Metabolites assessed were glucose, total cholesterol, urea, bilirubin, triglycerides, non-esterified fatty acids (NEFA), β-hydroxybutyrate (BHB) and creatinine. An aliquot of plasma was also used to determine insulin concentration using a commercial ELISA kit (Mercodia Insulin ELISA, Mercodia AB, Uppsala, Sweden), following the manufacturer’s instructions.

### RNA extraction, cDNA syntesis Target Genes, and Quantitative PCR

Total RNA was extracted from whole blood collected in PAXgene Blood RNA tubes following the manufacturer’s protocol (Blood RNA Kit Handbook; PreAnalytix GmbH, Qiagen, Hilden, Germany), as previously described [17]. RNA concentration was determined using the Qubit RNA BR Assay Kit (Invitrogen, Thermo Fisher Scientific, Waltham, MA, USA), and RNA integrity was assessed with the Agilent 4200 TapeStation System (Agilent Technologies, Waldbronn, Germany). The average RNA integrity value was 8.63 ± 0.39 (mean ± SD).

Each RNA sample was diluted to a final concentration of 100 ng/μL using nuclease-free water prior to reverse transcription. Complementary DNA (cDNA) synthesis was performed with the RevertAid RT Reverse Transcription Kit (Thermo Fisher Scientific, Waltham, MA, USA) according to the manufacturer’s instructions and as described by Cattaneo et al. [18]. The resulting cDNA was diluted 1:4 (vol/vol) in DNase/RNase-free water and stored at −80 °C until use.

Quantitative PCR (qPCR) was conducted as reported by Florida et al. [19] using a CFX384 Touch Real-Time PCR Detection System (Bio-Rad, Hercules, CA, USA). Each 10 μL reaction contained 4 μL of diluted cDNA and 6 μL of a mixture comprising 5 μL of 1 × SYBR Green Master Mix (Bio-Rad, Hercules, CA, USA), 0.4 μL each of 10 μM forward and reverse primers, and 0.2 μL of nuclease-free water. Thirty-seven genes were selected for transcriptomic analysis to capture a broad range of biological processes relevant to immune function, stress response, and metabolic adaptation in calves. The genes analyzed were: *CD14, CD16, MYD88, TLR2, TLR4, IRAK1, IRAK4, NFKB1, ITGAL, ITGAM, ITGB2, SELL, SELPLG, CX3CR1, IL1B, IL18, TNFA, IL8, CASP1, NLRP3, S100A8, MPO, LTF, MMP9, PRTN3, LCN2, IDO1, LYZ, SOD1, SOD2, ALOX5, ALOX15, PPARA, HSPA5, DDIT3, FOXO3A,* and *PRKCB.* Further details regarding primer information, sequences, and qPCR amplification efficiencies are available in the Supporting Information. Primer specificity was assessed by melt curve analysis, and only genes showing a single melting peak were included in the analysis. Amplicon size was verified by agarose gel electrophoresis, confirming a single product of the expected size for each gene.

The qPCR efficiency and quantification cycle (Cq) values were obtained using LinRegPCR (Version 2017.1), a software designed for the analysis of qPCR data obtained from SYBR green or other fluorescent dye-based assays. The software establishes baseline fluorescence, performs a baseline subtraction, and sets a window-of-linearity for calculating PCR efficiencies per sample.Using the mean PCR efficiency for each amplicon, the Cq value per sample, fluorescence threshold (set to determine Cq), and starting concentration per sample (expressed in arbitrary fluorescence units) were calculated.

Final data were normalized using the geometric mean of four internal control genes: *SDHA*, *YWHAZ, RPL13A* and *UXT*. The stability of the normalization factor for these internal control genes was assessed using GeNorm software, yielding a favorable pairwise variation of 0.11. No further stability improvement was observed with the addition of a fifth endogenous control gene (*ACTB*).

### Statistical analysis

Data were analyzed with repeated measures mixed models with the GLIMMIX procedure of SAS version 9.4 (SAS Institute Inc., Cary, NC). For each parameter, four structures of covariance (compound symmetry, autoregressive order 1, Toeplitz, or spatial power) were tested and the one with the lowest Akaike information criterion was retained in the model. The model included the fixed effects were ADG from 60 to 70 days of age (ADG; LOW and HIGH), sampling day (day; 60 and 70), and their interaction (ADG*day), whereas calves were included as random effect. Birth body weight was tested as a covariate but was not significant and was therefore removed from the final model. A total of 40 calves (n = 20 per group) were included, which exceeds sample sizes reported in comparable studies investigating immune and gene expression responses to weaning stress in calves [20,2]. Model assumptions were evaluated by inspection of residual plots to assess normality and homogeneity of variance; no major deviations were detected. Least squares means were compared using Tukey adjustment for multiple comparisons. Comparisons with P values less than 0.05 were reported as statistically significant. Those with values between 0.1 and 0.05 were discussed in terms of trend.

## Results

### Feed intake and Growth performance

**Fig 1:**
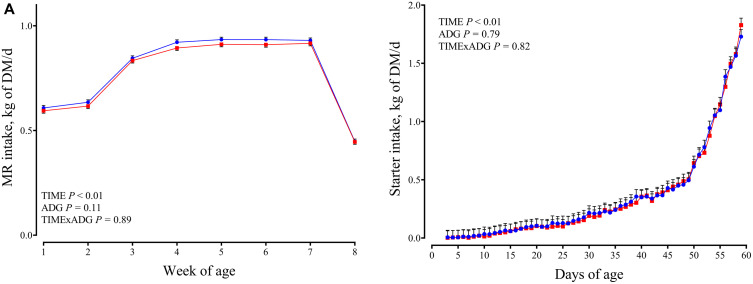
Milk and starter intake. Changes from 60 to 70 days of age in dairy calves with high average daily gain (HIGH; n = 20; blue circles) or low average daily gain (LOW; n = 20; red squares) in milk intake (kg of DM/d; A) and starter intake (kg of DM/d; **B)**. Data are presented as least squares means ± SEM.

During the pre-weaning period, milk intake did not differ between ADG classes (Fig 1). Similarly, starter intake was not affected by ADG class (0.338 vs. 0.332 kg/d; *P* = 0.79; Fig 1). No effects of time or time × ADG interaction were detected for either variable.

**Fig 2:**
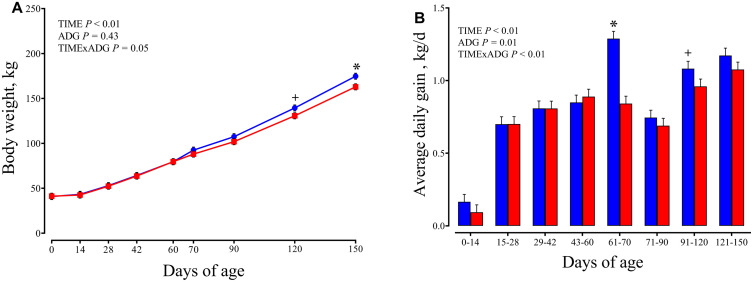
Growth performance. Changes from 60 to 70 days of age in dairy calves with high average daily gain (HIGH; n = 20; blue circles) or low average daily gain (LOW; n = 20; red squares) in body weight (kg; A) and average daily gain (kg/d; **B)**. Data are presented as least squares means ± SEM. Significance levels of the main effects of the models are reported. * indicates a significant difference between groups on the same day (P ≤ 0.05), and + indicates a tendency (0.05 < P < 0.10).

BW did not differ between ADG classes overall, but a significant ADG × time interaction was detected (P = 0.05; Fig 2). The BW remained similar between groups from birth to 90 days (P > 0.1). From 120 days onward, HIGH calves exhibited greater BW compared with LOW calves, with a tendency at 120 days (139.43 vs. 130.69 ± 2.245 kg; P = 0.01) and significant differences at 150 days (174.73 vs. 162.90 ± 2.245 kg; P < 0.01). Average daily gain (ADG) was different between ADG classes (P = 0.01), and a significant ADG × time interaction was observed (P < 0.01). As expected based on the classification criterion, ADG differed between groups at 70 days (1.29 vs. 0.84 ± 0.053 kg/d for HIGH vs. LOW; P < 0.01), because ADG class was defined using the growth rate from 60 to 70 days (Fig 2).

### Blood metabolites

**Fig 3:**
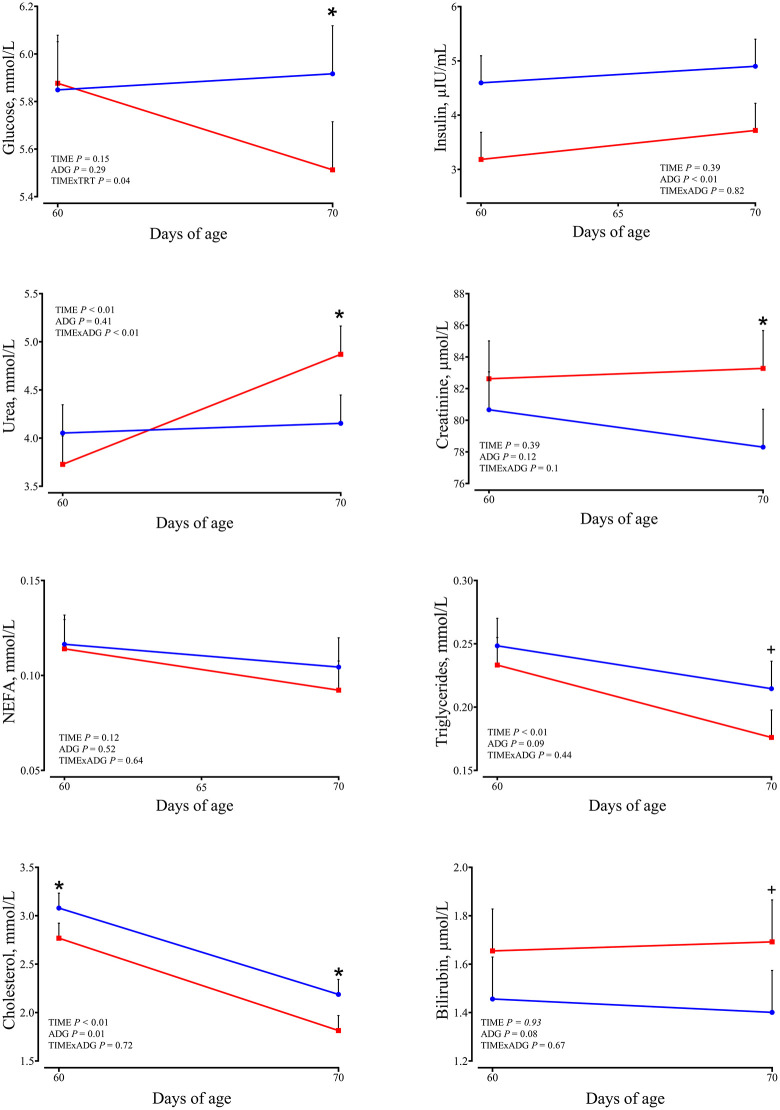
Plasma metabolite concentrations. Changes from 60 to 70 days of age in dairy calves with high average daily gain (HIGH; n = 20; blue circles) or low average daily gain (LOW; n = 20; red squares) in plasma metabolite concentrations. Data are presented as least squares means ± SEM. * indicates a significant difference between groups on the same day (P ≤ 0.05), and + indicates a tendency (0.05 < P < 0.10).

Plasma concentrations of circulating metabolites are presented in Fig 3.

Glucose showed a time × ADG interaction (*P* = 0.04). At 70 days of age, LOW calves exhibited lower glucose compared with HIGH calves (5.51 vs. 5.92 ± 0.202 mmol/L; *P* = 0.05). Insulin concentration was affected by ADG class (P < 0.01), with greater values in HIGH compared with LOW calves (4.75 vs. 3.45 ± 0.029 µIU/mL on average). Cholesterol concentration decreased from 60 to 70 days (*P* < 0.01). An effect of ADG class was detected (*P* = 0.01), with greater values in HIGH compared with LOW calves (3.08 vs. 2.77 ± 0.155 mmol/L at 60 days; *P* = 0.05, and 2.19 vs. 1.81 ± 0.155 mmol/L at 70 days; *P* = 0.02). Urea concentration increased over time (*P* < 0.01) and at 70 days, LOW calves showed greater concentrations compared with HIGH calves (4.87 vs. 4.15 ± 0.294 mmol/L; *P* = 0.02). BHB concentrations were not affected by time, ADG class, or their interaction (P > 0.10), with similar values observed in HIGH and LOW calves at both 60 and 70 days of age. Bilirubin concentration tended to be greater in LOW calves (*P* = 0.08). Triglyceride concentration decreased over time (*P* < 0.01) and tended to be greater in HIGH calves compared with LOW calves (*P* = 0.09). NEFA concentrations were not affected by time, growth class, or their interaction, with similar values in HIGH and LOW calves. Creatinine showed a tendency for a time × ADG interaction (P = 0.10). At 70 days of age, LOW calves exhibited greater concentrations compared with HIGH calves (83.28 vs. 78.30 ± 2.391 µmol/L; P = 0.04).

### Blood leucocytes Gene expression

**Fig 4:**
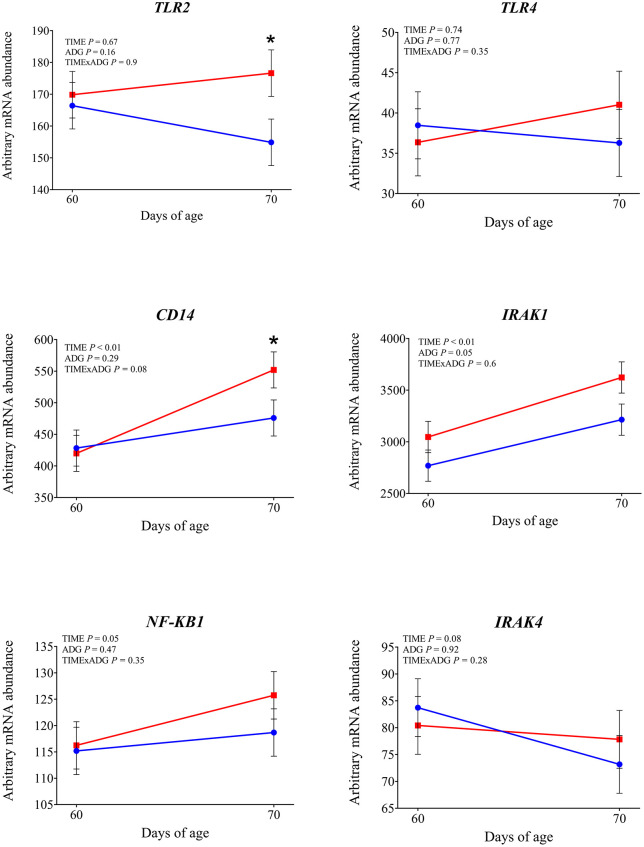
mRNA abundance of genes involved in TLR signaling and innate immune sensing. Changes from 60 to 70 days of age in dairy calves with high average daily gain (HIGH; n = 20; blue circles) or low average daily gain (LOW; n = 20; red squares) in arbitrary mRNA abundance (least squares means ± SEM) of genes involved in Toll-like receptor signaling and innate immune sensing in circulating blood leukocytes. * indicates a significant difference between groups on the same day (P ≤ 0.05), and + indicates a tendency (0.05 < P < 0.10).

**Fig 5:**
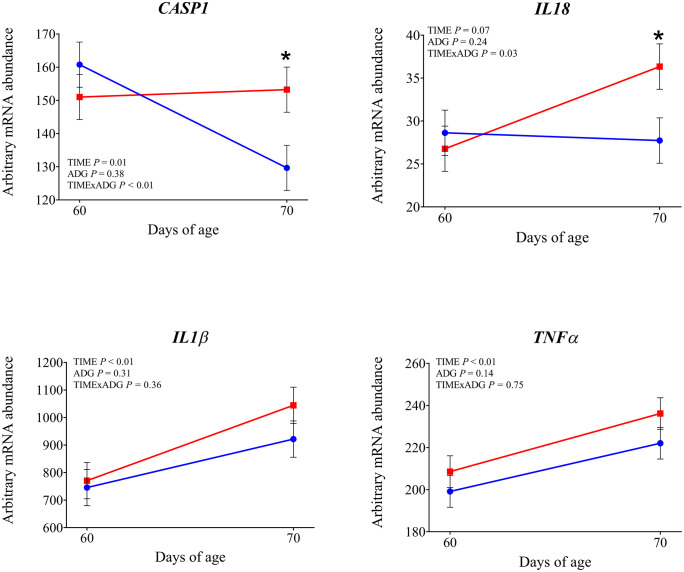
mRNA abundance of genes involved in inflammasome activation and inflammatory cytokines. Changes from 60 to 70 days of age in dairy calves with high average daily gain (HIGH; n = 20; blue circles) or low average daily gain (LOW; n = 20; red squares) in arbitrary mRNA abundance (least squares means ± SEM) of genes involved in inflammasome activation and inflammatory cytokine production in circulating blood leukocytes. * indicates a significant difference between groups on the same day (P ≤ 0.05), and + indicates a tendency (0.05 < P < 0.10).

**Fig 6:**
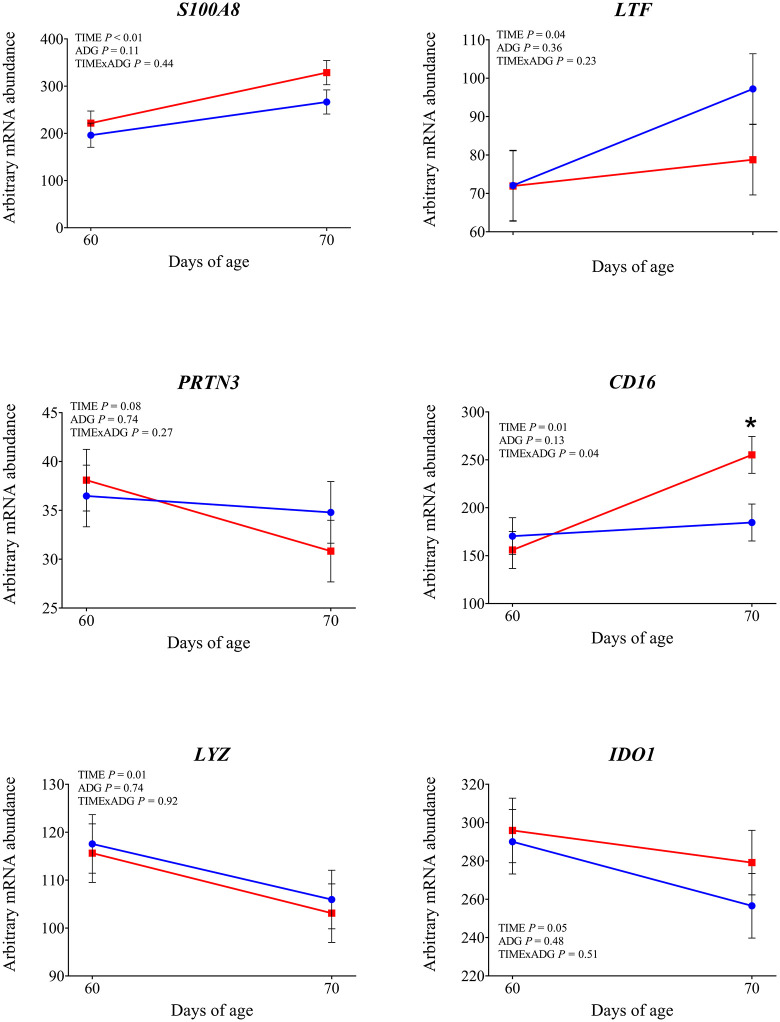
mRNA abundance of genes involved in neutrophil activation and antimicrobial responses. Changes from 60 to 70 days of age in dairy calves with high average daily gain (HIGH; n = 20; blue circles) or low average daily gain (LOW; n = 20; red squares) in arbitrary mRNA abundance (least squares means ± SEM) of genes involved in neutrophil activation and antimicrobial responses in circulating blood leukocytes. * indicates a significant difference between groups on the same day (P ≤ 0.05), and + indicates a tendency (0.05 < P < 0.10).

**Fig 7:**
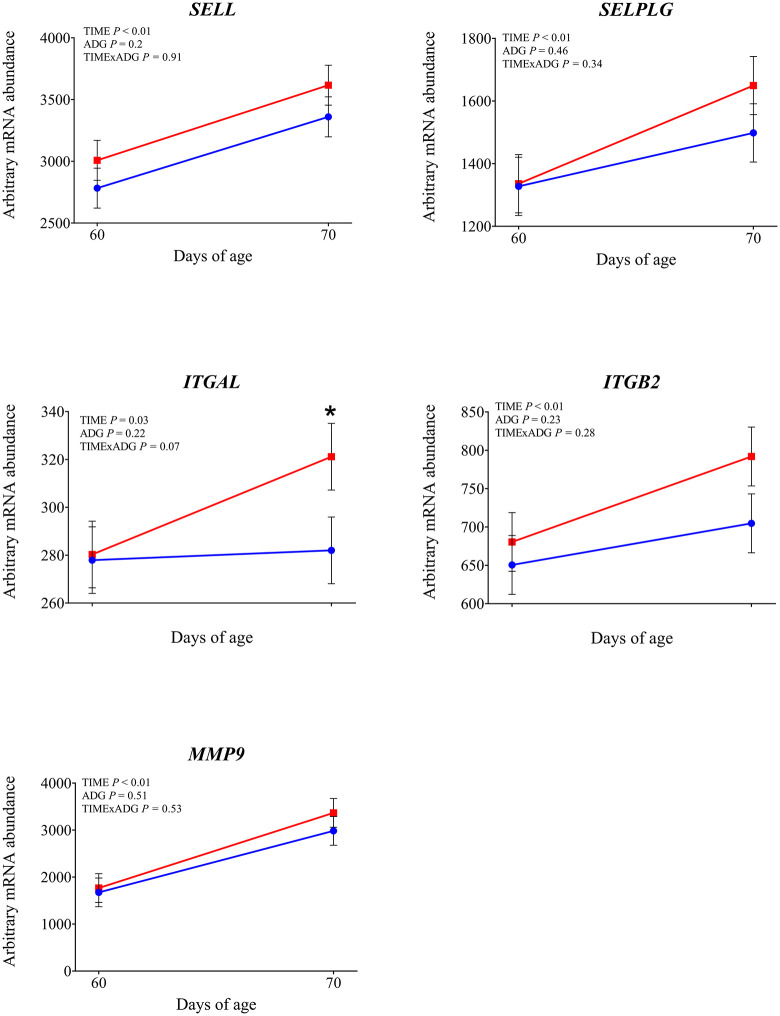
mRNA abundance of genes involved in leukocyte adhesion and cell trafficking. Changes from 60 to 70 days of age in dairy calves with high average daily gain (HIGH; n = 20; blue circles) or low average daily gain (LOW; n = 20; red squares) in arbitrary mRNA abundance (least squares means ± SEM) of genes involved in leukocyte adhesion and cell trafficking in circulating blood leukocytes. * indicates a significant difference between groups on the same day (P ≤ 0.05), and + indicates a tendency (0.05 < P < 0.10).

**Fig 8:**
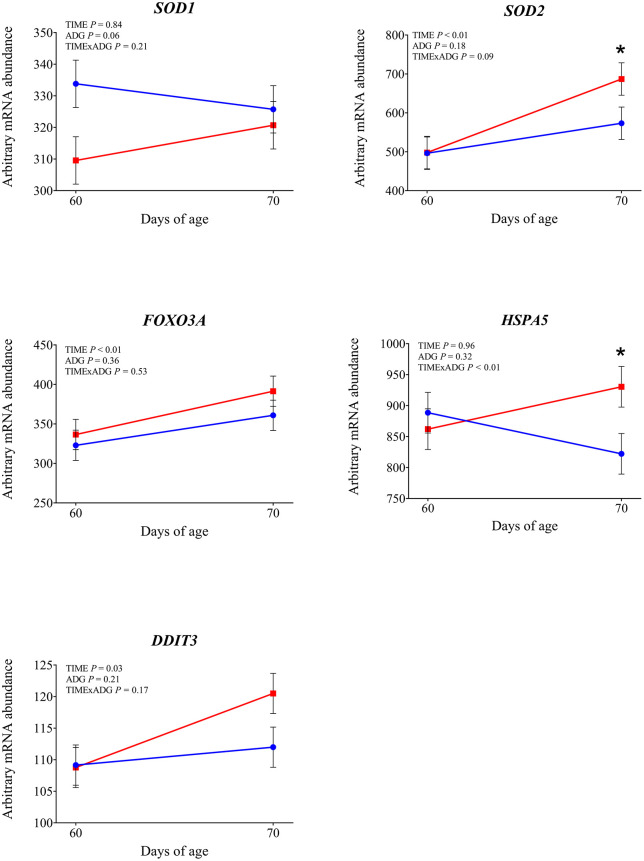
mRNA abundance of genes involved in cellular stress responses. Changes from 60 to 70 days of age in dairy calves with high average daily gain (HIGH; n = 20; blue circles) or low average daily gain (LOW; n = 20; red squares) in arbitrary mRNA abundance (least squares means ± SEM) of genes involved in cellular stress responses in circulating blood leukocytes. * indicates a significant difference between groups on the same day (P ≤ 0.05), and + indicates a tendency (0.05 < P < 0.10).

**Fig 9:**
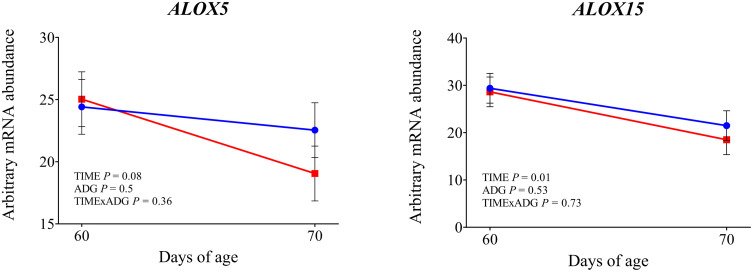
mRNA abundance of genes involved in lipid mediator metabolism. Changes from 60 to 70 days of age in dairy calves with high average daily gain (HIGH; n = 20; blue circles) or low average daily gain (LOW; n = 20; red squares) in arbitrary mRNA abundance (least squares means ± SEM) of genes involved in lipid mediator metabolism in circulating blood leukocytes. * indicates a significant difference between groups on the same day (P ≤ 0.05), and + indicates a tendency (0.05 < P < 0.10).

The mRNA abundance of genes investigated in the whole blood leukocytes is shown in Fig 4–9 and in the Supporting Information (S2 Table).

Expression of *TLR2* tended to be affected by time × ADG (*P* = 0.10). At 70 days of age was observed a significantly higher expression in LOW calves compared with those with HIGH calves (*P* = 0.04). The expression of *TLR4* was not affected by time, ADG, or their interaction (*P* > 0.10). Among the genes involved in intracellular signal transduction, *IRAK1* showed a significantly greater expression at 70 days of age compared with 60 days (*P* < 0.01). In addition LOW animals tended to have higher expression compared to the high ADG calves (*P* = 0.05). Similarly, *IRAK4* tended to increase over time (*P* = 0.08). No significant effects were found for *MYD88* (*P* > 0.10). Regarding the transcriptional response, *NFKB1* expression increased after weaning (*P* = 0.05), while *CD14*, a co-receptor involved in LPS recognition, was also significantly upregulated at 70 days of age (*P* < 0.01), independently of ADG. *CD16* showed a significant time × ADG interaction (*P* = 0.04). At 70 days of age, expression was higher in calves with low ADG compared with high ADG (*P* = 0.01). Expression of *PRKCB*, a gene involved in intracellular signaling downstream of TLR activation, was not affected by time, ADG class, or their interaction (*P* > 0.10). Expression of *SELL* and *SELPLG* was significantly higher at 70 d compared with 60 d (*P* < 0.01). Among the integrin subunits, *ITGB2* was upregulated at 70 days (*P* < 0.01), while *ITGAL* also showed a significant time effect (*P* = 0.03). A tendency for a time × ADG interaction was observed for *ITGAL* (*P* = 0.07), and at 70 days, expression was higher in low-ADG calves compared with high-ADG calves (*P* = 0.05). Expression of ITGAM was not affected by time, ADG, or their interaction (*P* > 0.10). Expression of *CX3CR1* was not affected by time, ADG, or time × ADG interaction (*P* > 0.10). Expression of genes involved in antimicrobial functions *MMP9, S100A8*, *LTF*, and *LYZ* was significantly higher at 70 days compared with 60 days (P < 0.05). A tendency for increased expression over time was observed for *PRTN3* (*P* = 0.08) and *IDO1* (*P* = 0.05). The expression of *MPO*, *LCN2*, and *CX3CR1* was not affected by time, ADG, or their interaction (*P* > 0.10). Expression of *IL1β* and *TNFα* was significantly higher at 70 days compared with 60 days (*P* < 0.01). Expression of *CASP1* also increased after weaning (*P* = 0.01), with a significant time × ADG interaction (*P* < 0.01). Low-ADG calves showed higher expression compared with high-ADG calves at 70 days (*P* = 0.02). Expression of *IL18* tended to increase over time (*P* = 0.07), and a significant time × ADG interaction was detected (*P* = 0.03). At 70 days, expression was significantly higher in low-ADG calves compared with high-ADG calves (*P* = 0.02). Expression of *IL8* and *NLRP3* was not affected by time, ADG class, or their interaction (P > 0.10). Expression of *SOD2*, which encodes the mitochondrial superoxide dismutase, increased significantly at 70 days compared with 60 days (*P* < 0.01). A tendency for higher expression in low-ADG calves was also observed (*P* = 0.05). For *SOD1*, a tendency for an ADG effect was detected (*P* = 0.06), with significantly higher expression in high-ADG calves at 60 days (*P* = 0.03), and no difference at 70 days (*P* = 0.64). Among genes involved in leukotriene metabolism, *ALOX15* expression significantly decreased over time (*P* < 0.01), with no differences between ADG classes or interaction effects. *ALOX5* showed a tendency to decrease over time (*P* = 0.08). Expression of PPARA was not affected by time, ADG class, or their interaction (*P* > 0.10). Expression of *FOXO3A* was greater at 70 days compared with 60 days (*P* < 0.01). Expression of *DDIT3* also increased over time (*P* = 0.03). Expression of *HSPA5* was affected by a time × ADG interaction (*P* < 0.01). At 70 days, calves with low ADG showed higher expression compared with high ADG (*P* = 0.02).

## Discussion

Weaning represents a multifactorial stressor as calves simultaneously encounter changes in diet, housing, and social structure [2, 3, 7]. These stress induced responses that can impair growth and increase disease susceptibility during early life [1]. Within this context, we investigated how this transition modulates immune-related gene expression in circulating leukocytes, and whether calves with divergent growth performance exhibit distinct immunometabolic responses.

### Effect of weaning on immune-related gene expression

The changes in expression observed across a broad set of immune-related genes suggest that calves experienced a measurable shift in innate immune activation after weaning. A first notable change was the upregulation of genes related to microbial recognition and signal transduction, including *CD14, IRAK1*, and *NFKB1*. Although *TLR2* and *TLR4* did not change significantly, the increase in *CD14*, an amplifier of TLR signaling, suggests enhanced leukocyte sensitivity to LPS and other danger signals [21]. Similar modulation of TLR-related pathways following weaning or other management stressors has been described in beef and dairy calves [20,22]. The TLRs engagement triggers NF-κB–mediated transcription of pro-inflammatory cytokines [23], which is consistent with increased expression of pro-inflammatory cytokines *IL1β* and *TNFA* after weaning. Similar increases in *IL-1* family cytokines and *TNF-α* have been reported by O’Loughlin et al., [20] who observed upregulation of *IL-1β, IL-8, IFN-γ*, and *TNF-α* in leukocytes for several days after weaning, confirming that this stress can enhance innate immune activation. The upregulation of *CASP1* and *IL18* further supports the involvement of inflammasome-associated pathways, known to mediate the maturation and release of *IL-1* family cytokines during immune activation after weaning, in both blood and mucosal immune cells (O’Loughlin et al., 2011; Lynch et al., 2010).

Weaning altered the expression of genes involved in leukocyte adhesion and trafficking. In our study, *SELL* and *SELPLG*, encoding L-selectins critical for leukocyte rolling, were both upregulated, as were *ITGAL* and *ITGB2*, which form the β2-integrin complex necessary for firm adhesion and transmigration [24]. These transcriptional changes are consistent with increased myeloid mobilization previously observed after weaning [2], although surface expression of adhesion molecules can vary depending on stress intensity and context [25–27]. Our gene expression data suggest an increased potential for leukocyte extravasation and immune cell trafficking. Supporting this, we found higher expression of *MMP9*, a matrix metalloproteinase involved in extracellular matrix degradation, which facilitates neutrophil migration toward sites of inflammation [28], and of *S100A8* and *LTF*, markers of activated neutrophils. Although some antimicrobial transcripts such as *LYZ* and *PRTN3* showed no change or a downward trend, the overall pattern suggests enhanced myeloid readiness. Weaning appeared to also influence oxidative and metabolic stress responses. *SOD2* was upregulated, suggesting elevated ROS detoxification activity during or after immune activation. Likewise, *FOXO3A* and *DDIT3*, both involved in stress response regulation, were highly expressed post-weaning. These findings imply that leukocytes not only become immunologically activated, but also experience increased metabolic and redox demands. The decrease of *ALOX5* and *ALOX15* indicate changes in lipid mediator pathways. Taken together, these findings suggest that weaning may trigger a coordinated activation of innate immune, adhesion, and stress-related pathways in circulating leukocytes.

### Post-weaning immune and metabolic markers in LOW and HIGH growth rate calves

Given the relevance of early-life growth for future productive performance [29], we evaluated whether post-weaning growth performance was associated with distinct immune and metabolic responses in circulating leukocytes.

Specifically, *TLR2*, *IRAK1* and *CD14* expression was greater in LOW than in HIGH calves at 70 days. This results may indicates that, after weaning, LOW calves maintained a more reactive state of innate immune surveillance. Weaning is known to induce TLR-related immune activation in calves [2,20], and the heightened transcriptional response in LOW calves suggests a more pronounced immune activation in response to the stress associated with weaning. This greater signaling in LOW calves was accompanied by greater activation of the inflammasome pathway, as indicated by higher expression of *CASP1* and *IL18* at 70 days. *IL18* promotes interferon-γ production and the activation of natural killer cells and T-helper 1 lymphocytes [30] and its higher expression in LOW calves likely reflects a more sustained inflammatory response following weaning. This interpretation is reinforced by the higher expression of *CD16* in LOW calves, a receptor commonly expressed on activated neutrophils and monocytes, which mediates the clearance of antigen–antibody complexes from the circulation. The LOW calves also showed transcriptional evidence of greater leukocyte adhesion and trafficking capacity. The gene *ITGAL* was more expressed in LOW calves at 70 days, suggesting increased leukocyte extravasation potential. The changes observed in LOW calves are consistent with post-weaning neutrophil activation reported in the literature [2,20,31] suggesting that such changes may have been more pronounced in LOW calves. Expression of *HSPA5* was higher in LOW calves at 70 days, indicating a higher level of endoplasmic reticulum stress after weaning. Similarly, *SOD2* was higher in LOW animals, suggesting a greater mitochondrial oxidative load in these calves. The combined upregulation of *HSPA5* and *SOD2* therefore supports the interpretation that LOW calves faced higher cellular and metabolic strain after weaning. Taken together, these findings support the idea that LOW calves experienced a greater inflammatory response to weaning.

Metabolic biomarkers confirm the interpretation of a less favourable condition in LOW calves. Insulin concentrations were lower in LOW calves at both 60 and 70 days. Although insulin levels diverged between groups at 60 days, this occurred independently of glucose availability, and the biological meaning remains difficult to define. After weaning, however, LOW calves displayed both lower insulin and lower glucose. This combination may indicates reduced circulating energy availability in LOW animals during the post-weaning and is consistent with a greater metabolic demand associated with their grater activation of innate immune pathways [32,33]. In this context, part of the available glucose may have been partially redirected toward supporting immune function rather than growth in LOW calves, although differences in nutrient intake cannot be excluded as a contributing factor. Urea concentrations were higher in LOW calves after weaning. Since calves were housed in group pens and individual feed intake could not be measured, we cannot entirely rule out the possibility that differences in urea reflected variation in starter intake. However, creatinine, a marker of protein turnover, was also higher in LOW calves, suggesting increasing mobilization of muscle. The greater concentration of urea and creatinine support the idea that LOW calves relied more heavily on amino acid oxidation to cope with the greater energetic demands imposed by immune activation and adaptation to weaning compared to the HIGH calves. One limitation of the present study is that individual feed intake was not measured after weaning, when calves were group-housed. Therefore, differences in nutrient intake between groups cannot be excluded as a contributing factor to the observed metabolic responses. BHB, an indirect marker of solid feed intake and rumen development [34], was evaluated. The lack of differences in BHB between groups suggests that variation in solid feed intake alone may not fully explain the observed responses.

Differences in plasma lipid markers between LOW and HIGH calves provided additional metabolic indicators that may have influenced their distinct growth outcomes. Despite the absence of significant differences in NEFA between groups, triglyceride concentrations were lower in LOW calves. This pattern suggests that the reduction in circulating triglycerides was more likely attributable to decreased intestinal lipid absorption, rather than lipid mobilization. The hypothesis of altered lipid absorption is consistent with the findings of Wood et al. [35] who reported that abrupt dietary transitions during weaning can increased gastrointestinal permeability following weaning, a change that could plausibly impact nutrient absorption, including lipids. The gastrointestinal tract is a major immunological interface during early life [36], and the increased expression of *TLR2*, *CASP1*, and *IL18* in LOW calves may reflect heightened exposure to luminal microbial signals due to increased intestinal permeability. Increased intestinal permeability following weaning may allow for the translocation of microbial products such as LPS, potentially triggering TLR and inflammasome activation both locally and systemically [35,36].

Total cholesterol concentrations were also lower in LOW calves at both 60 and 70 days. Insulin is a known stimulator of hepatic cholesterol synthesis [37], and the consistently lower insulin concentrations observed in LOW calves are compatible with the lower concentration of cholesterol. Additionally, bilirubin concentrations were elevated in LOW calves, potentially reflecting increased hepatic metabolic load or reduced liver clearance capacity. Bilirubin is commonly used as a surrogate marker for hepatic function [38], and its greater level is consistent with the overall pattern of altered metabolic status observed in these animals.

## Conclusion

Weaning represents a critical transition in calf development, involving dietary, environmental, and social changes that trigger significant immunometabolic adaptations. In this study, we observed measurable activation of innate immune pathways and stress responses following weaning, confirming its role as a physiological challenge for all animals. However, calves with lower post-weaning growth showed stronger immune activation and distinct metabolic changes. One set of changes suggested a shift in energy allocation: lower glucose and insulin levels, together with higher urea and creatinine, were consistent with a shift in nutrient utilization toward immune-related processes rather than growth, and that muscle protein was likely used to meet increased energy demands, although differences in nutrient intake cannot be excluded. A second set of findings pointed to altered nutrient assimilation. Despite no difference in NEFA levels, LOW calves had lower triglycerides, suggesting impaired lipid absorption, possibly linked to increased intestinal permeability after weaning. This may have further limited energy availability. Together, these results show that weaning imposes a metabolic burden on calves, but individual capacity to cope with this stress varies. Understanding these differences may help identify animals at risk of poor growth and guide nutritional strategies to improve early adaptation and long-term productivity.

## Supporting information

S1 Table Primers used for qPCR. GenBank accession number, hybridization position, sequence, amplicon size and source of primers for Bos taurus used to analyze gene expression by qPCR.
(DOCX)

S2 Table mRNA abundance. Arbitrary mRNA abundance for gene expression in circulating blood leucocytes in calves with high ADG (HIGH; n = 20), or low ADG (LOW; n = 20) from 60 to 70 days of age.
(DOCX)
