# Supplementary material for: Circulating leukocyte gene expression responses to weaning and their association with growth in holstein calves
Source: PLoS One. 2026 May 27;21(5):e0349643. doi: 10.1371/journal.pone.0349643 (PMC13215499; doi:10.1371/journal.pone.0349643)
Supplement: S1 Table — GenBank accession number, hybridization position, sequence, amplicon size and source of primers for Bos taurus used to analyze gene expression by qPCR. (DOCX) [file pone.0349643.s001.docx]

| **S1. Primers used for qPCR.** GenBank accession number, hybridization position, sequence, amplicon size and source of primers for Bos taurus used to analyze gene expression by qPCR. | | | | |
| --- | --- | --- | --- | --- |
| **Accession no.** | **Gene** | **Primers^1^** | **Primers (5’-3’)** | **(bp)^2^** |
| NM_198221.2 | *ITGAL* | F.1582 | ATCAACGGGGATGAGCTGAC | 126 |
|  |  | R.1707 | GGTCCCTTCTATCCGCTGAC |  |
| NM_175781.1 | *ITGB2* | F.1231 | GACACCCTGAAAGTCACCTACGA | 108 |
|  |  | R.1338 | GAAGGTGATCGGGACGTTGAT |  |
| NM_174744.2 | *MMP9* | F.1169 | GCCCGGATCAAGGATACAGC | 128 |
|  |  | R.1296 | GGGGTGCTCCTCTGTGAATC |  |
| NM_174182.1 | *SELL* | F.588 | CTCTGCTACACAGCTTCTTGTAAACC | 104 |
|  |  | R.691 | CCGTAGTACCCCAAATCACAGTT |  |
| NM_001037628.2 | *SELPLG* | F.123 | CTGAGCACGGTGCCATGTTTC | 111 |
|  |  | R.233 | CTGGGGCCTTCACAGTTTCA |  |
| NM_174008.1 | *CD14* | F.525 | TCCGTAACGTATCGTGGACAAC | 100 |
|  |  | R.624 | GAGTGTGCTTGGGCAATGTTC |  |
| XM_003586675.4 | *LCN2* | F.749 | CCAGTGAGCCTGCACCTTTG | 141 |
|  |  | R.889 | TATTTAGCAGGCAAGGCAGGG |  |
| NM_001113298.2 | *MPO* | F.1311 | AGCCATGGTCCAGATCATCAC | 105 |
|  |  | R.1415 | ACCGAGTCGTTGTAGGAGCAGTA |  |
| NM_174615.2 | *SOD1* | F.256 | GGCTGTACCAGTGCAGGTCC | 101 |
|  |  | R.356 | GCTGTCACATTGCCCAGGT |  |
| NM_201527.2 | *SOD2* | F.620 | TGTGGGAGCATGCTTATTACCTT | 95 |
|  |  | R.714 | TGCAGTTACATTCTCCCAGTTGA |  |
| NM_174197.2 | *TLR2* | F.3182 | CCATGTCTGGAGAGGGTGTT | 102 |
|  |  | R.3283 | GGGGACACAAAACAGCACTT |  |
| NM_174178.2 | *SDHA* | F.1563 | CTGAAGCAGGTTTCAACACG | 130 |
|  |  | R.1544 | GTTGTCCTCCTCCATGTTCC |  |
| NM_173925.2 | *IL8* | F.350 | GTGAAGAGAGCTGAGAAGCAAG | 150 |
|  |  | R.499 | CACCAGACCCACACAGAACAT |  |
| NM_001075998.1 | *IRAK4* | F.54 | CGCCCGGGCAGGAATAAAAT | 133 |
|  |  | R.186 | GCGACTGCTAACTTCTTCCATC |  |
| NM_001076998.2 | *RPL13A* | F.417 | CTCGCAAGTTTGCCTACCTA | 113 |
|  |  | R.529 | CCGATAGTGGATCTTGGCCT |  |
| NM_174198.6 | *TLR4* | F.102 | GCTGTTTGACCAGTCTGATTGC | 102 |
|  |  | R.203 | GGGCTGAAGTAACAACAAGAGGAA |  |
| NM_001078159.1 | *LYZ* | F.281 | AAAGCAGTTAACGCCTGTCGTAT | 122 |
|  |  | R.402 | CATGCCACCCATGCTTTAATG |  |
| NM_180998.2 | *LTF* | F.631 | CCAGGGAGCTGTGGCTAAAT | 135 |
|  |  | R.765 | AAGTATGGTTCCCGGGAGGA |  |
| NM_001102219.1 | *NLRP3* | F.69 | CTTTCTGGACTCTGACCGGG | 149 |
|  |  | R.217 | ATTGAGGTGCAGCCCTTCTG |  |
| NM_001040555.1 | *IRAK1* | F.950 | CCTCAGCGACTGGACATCCT | 103 |
|  |  | R.1052 | GGACGTTGGAACTCTTGACATCT |  |
| NM_008361.4 | *IL1B* | F.120 | GCCACCTTTTGACAGTGATGAG | 137 |
|  |  | R.256 | TGATGTGCTGCTGCGAGATT |  |
| NM_001077402.1 | *CD16* | F.252 | CAGGACAGTGGCGAGTACAAGT | 100 |
|  |  | R.351 | GAGCGACCTGGAGCAATAGC |  |
| NM_001014382.2 | *MYD88* | F.367 | GGAGGACTGCCAAAAGTATATTCTG | 105 |
|  |  | R.471 | GCCATGTCATTTATCCGAGTTATG |  |
| NM_001113725.2 | *S100A8* | F.19 | ATTTTGGGGAGACCTGGTGG | 124 |
|  |  | R.142 | ACGGCGTGGTAATTCCCTTT |  |
| NM_001101866.2 | *IDO1* | F.18 | ACTGCAAGAATGGCAGGTGA | 125 |
|  |  | R.142 | GGATGAGGTAGGTCCTCCAGT |  |
| XM_592026.7 | *CASP1* | F.193 | AGTGCTGAACCAGGAGGAGA | 188 |
|  |  | R.380 | CAGACTGTGAACCTGAAGTGAG |  |

| **S1. Primers used for qPCR.** GenBank accession number, hybridization position, sequence, amplicon size and source of primers for Bos taurus used to analyze gene expression by qPCR. | | | | |  |
| --- | --- | --- | --- | --- | --- |
| **Accession no.** | **Gene** | **Primers^1^** | **Primers (5’-3’)** | **(bp)^2^** |  |
| NM_001192792 | *ALOX5* | F.771 | GCAGGAAGACCGCATGTTTG | 163 |  |
|  |  | R.933 | GTTCCCTTGCTCGATCTCCT |  |  |
| NM_174501.2 | *ALOX15* | F.1495 | AGGCCTGGTGTCGAGATATCA | 105 |  |
|  |  | R.1599 | TGGTCACAAAGTGGCAAAGC |  |  |
| NM_174091.2 | *IL18* | F.366 | GACTGTTCAGATAATGCACCCC | 126 |  |
|  |  | R.491 | GTTCTCACAGGAGAGAGTAGAC |  |  |
| NM_173966.3 | *TNFα* | F.174 | CCAGAGGGAAGAGCAGTCCC | 114 |  |
|  |  | R.287 | TCGGCTACAACGTGGGCTAC |  |  |
| NM_174814.2 | *YWHAZ* | F. | TGAAAATGAAAGGAGACTACTACCG | 84 |  |
|  |  | R. | GCTGTGACTGGTCCACAATC |  |  |
| NM_001039957.1 | *ITGAM* | F.268 | GGCTTGTCTCTTGCATTTGCT | 95 |  |
|  |  | R.362 | CCATTTGCATAGGTGTTCTCCTT |  |  |
| NM_001102558.2 | *CX3CR1* | F.75 | CCCAGCCCAGGTGCTCA | 175 |  |
|  |  | R.249 | CAGCAAATTTCCCACCAGGC |  |  |
| XM_002689143 | *PRTN3* | F.222 | TGCCTGAACAACTTGAACCC | 108 |  |
|  |  | R.329 | CTCAAACAGGCGACTGATGC |  |  |
| NM_001034036.1 | *PPARα* | F.581 | ATGTGAGGGCTGCAAGGGTT | 172 |  |
|  |  | R.752 | TCGTCCAAAACGAATCGCGT |  |  |
| NM_001075148.1 | *HSPA5* | F.998 | GTAGAAAAGGCCAAACGGGC | 145 |  |
|  |  | R.1142 | TCATGGTGGAACGGAACAGG |  |  |
| NM_001078163.1 | *DDIT3* | F.319 | CTTCACCACTCTTGACCCCG | 115 |  |
|  |  | R.433 | CAGGGAGCTCTGACTGGACT |  |  |
| NM_001206083.1 | *FOXO3A* | F.605 | CTGCCGGCTGGAAGAACTCTAT | 182 |  |
|  |  | R.786 | GCTCTTGGTGTACTTGTTGCTG |  |  |
| NM_174587.1 | *PRKCB* | F.223 | CACCGACTTCATCTGGGGCT | 191 |  |
|  |  | R.413 | CACCGACTTCATCTGGGGCT |  |  |
| NM_001037471.2 | *UXT* | F. | TGTGGCCCTTGGATATGGTT |  |  |
|  |  | R. | GGTTGTCGCTGAGCTCTGTG |  |  |
|  | *NFKB1* | F.172 | TTCAACCGGAGATGCCACTAC | 95 |  |
|  |  | R.266 | ACACACGTAACGGAAACGAAATC |  |  |
| ^1^ Primer direction (F – forward; R – reverse) and hybridization position on the sequence. are underlined.  ^2^ Amplicon size in base pair (bp). | | | | | |
