# Supplementary material for: Circulating leukocyte gene expression responses to weaning and their association with growth in holstein calves
Source: PLoS One. 2026 May 27;21(5):e0349643. doi: 10.1371/journal.pone.0349643 (PMC13215499; doi:10.1371/journal.pone.0349643)
Supplement: S2 Table — Arbitrary mRNA abundance for gene expression in circulating blood leucocytes in calves with high ADG (HIGH; n = 20), or low ADG (LOW; n = 20) from 60 to 70 days of age. (DOCX) [file pone.0349643.s002.docx]

**S2. mRNA abundance.** Arbitrary mRNA abundance for gene expression in circulating blood leucocytes in calves with high ADG (HIGH; n = 20), or low ADG (LOW; n = 20) from 60 to 70 days of age.

|  | | | | *P*-value | | | |
| --- | --- | --- | --- | --- | --- | --- | --- |
| Gene | HIGH | LOW | SEM | | TIME | ADG | TIMExADG |
| *LCN2* | 54.98 | 56.64 | 3.702 | | 0.72 | 0.72 | 0.25 |
| *NLRP3* | 159.34 | 166.90 | 10.431 | | 0.45 | 0.56 | 0.23 |
| *MYD88* | 117.91 | 135.76 | 9.614 | | 0.14 | 0.14 | 0.84 |
| *MPO* | 190.91 | 198.31 | 18.452 | | 0.19 | 0.69 | 0.11 |
| *CX3CR1* | 202.52 | 211.3 | 12.35 | | 0.99 | 0.56 | 0.78 |
| *PPARα* | 38.75 | 41.67 | 2.03 | | 0.56 | 0.25 | 0.5 |
| *PRKCB* | 205.54 | 204.64 | 7.163 | | 0.73 | 0.91 | 0.36 |
| *ITGAM* | 160.61 | 166.40 | 8.537 | | 0.46 | 0.58 | 0.65 |
| *IL8* | 143.64 | 114.64 | 19.353 | | 0.11 | 0.22 | 0.82 |

^Data are presented as mean and SEM. Significance levels of the main effects of the models are reported.^

^* Indicates a significant difference (P ≤ 0.05), + Indicates a trend (P < 0.1).^
